# The Expression of *STAT3* and *STAT5A* Genes in Severe Refractory Asthma

**Published:** 2017

**Authors:** Kayvan Saeedfar, Mehrdad Behmanesh, Esmaeil Mortaz, Mohammad Reza Masjedi

**Affiliations:** 1 Department of Genetics, Faculty of Biological Sciences, Tarbiat Modares University, Tehran, Iran; 2 Chronic Respiratory Diseases Research Center (CRDRC), National Research Institute of Tuberculosis and Lung Diseases (NRITLD), Shahid Beheshti University of Medical Sciences, Tehran, Iran; 3 Tobacco Control Research Center, Tehran, Iran.

**Keywords:** Asthma, Severe Asthma, *STAT3*, *STAT5a*, Gene Expression

## Abstract

**Background:**

Despite being a high burden disorder, the pathogenesis of severe refractory asthma (SRA) is poorly understood. There are some evidences for the involvement of members of the signal transducer and activator of transcription (STAT) family, including *STAT3* and *STAT5a*. Our study aimed to evaluate the gene expression of *STAT3* and *STAT5a* in asthma and SRA to establish if there is an association.

**Materials and Methods:**

Using quantitative real-time polymerase chain reactions (qRT-PCR), the transcript levels of *STAT3* and *STAT5a* were evaluated in peripheral blood mononuclear lymphocytes (PBML) isolated from 13 patients with SRA, 14 with mild asthma, and 30 healthy volunteers.

**Results:**

There were no significant differences in *STAT3* transcript levels between study groups. There was however a significant difference in *STAT5a* transcript levels between cases and controls (p-value=0.03). In comparison to healthy controls, the levels of *STAT5a* were notably lower in patients with mild asthma and significantly least in those with SRA.

**Conclusion:**

Our study found no appreciable association between *STAT3* gene expression and either mild asthma or SRA. However, the *STAT5a* down regulation in asthmatics and especially SRA is a notable finding which denotes on association between *STAT5a* and different level of asthma.

## INTRODUCTION

Chronic asthma is a non-communicable inflammatory airway disorder in which patients present with recurring bouts of breathlessness and wheezing. Although the exact pathogenesis of asthma is not fully understood, numerous causative environmental and/or triggering agents have been described. These include allergens, tobacco smoke, chemical irritants, and microorganisms. These factors interact with an individual’s genetic and epigenetic background, leading to the development of asthma or the triggering of attacks (1).

Asthma is suspected to cause approximately 250,000 premature deaths annually and the World Health Organization (WHO) estimates that more than 300 million individuals are affected worldwide. This number is predicted to increase to 400 million by 2025. Due to the fact that asthma is a major cause of disability, poor quality of life, increased health resource utilization, and is a public health concern, it is essential to fully understand the genetic basis of the disease (2, 3).

During previous decades, there has been some controversy concerning the definition and classification of severe refractory asthma (SRA), found in approximately in 5–10% of cases (4). In 2011, an international consensus statement was published by the Innovative Medicine Initiative (IMI) that aimed to clarify a unique definition, classification, and diagnostic algorithm for SRA. This stated that “the term ‘severe refractory asthma’ should be reserved for patients with asthma in whom alternative diagnoses have been excluded, comorbidities have been treated, trigger factors have been removed (if possible) and compliance with treatment has been checked, but still have poor asthma control or frequent (≥2) severe exacerbations per year despite the prescription of high-intensity treatment or can only maintain adequate control when taking systemic corticosteroids and are thereby at risk of serious adverse effects of treatment” (2).

In order to understand the fundamental mechanisms of asthma pathogenesis, efforts have been made to identify genetic associations with asthma and SRA (5,6). Among the numerous genes known to associate with asthma, members of the signal transducer and activator of transcription (STAT) pathway appear to have an important function (7). Seven proteins of this family have been shown to have roles in signal transduction pathways and/or gene transcription (8) and may be involved with the mechanisms that underlie asthma and SRA.

The gene coding for *STAT3* is located in chromosomal region 17q21.2 and consists of 24 exons. Through alternative splicing, *STAT3* can be expressed as three different splice variants. *STAT3* is an essential protein involved in cell growth and apoptosis, and is expressed in all tissue types. Furthermore, it can act as a transcription factor and may also be a co-activator of signal transduction by glucocorticoid receptors (9, 10).

Like *STAT3*, *STAT5* is related to glucocorticoid receptors and acts as a transcription activator in the immune system (11). There are two isoforms of *STAT5* (*STAT5a* and *STAT5b*), coded by two separate genes located at inverted positions within the 17q21.2 or 17q11.2 genomic regions (12,13). Several important cellular processes are influenced by *STAT5* isoforms, including replication, apoptosis, differentiation, and inflammation. It has also been shown that *STAT5a/b* are important for lymphocyte proliferation, apoptosis, and have been used for targeted gene therapy and therapeutics (e.g., for asthma and cancer) (14–17).

There is some evidence supporting the involvement *STAT3* and *STAT5* in the development of asthma (18, 19). *STAT3* has been demonstrated to be involved in airway inflammation, allergy, and asthma through several proposed mechanisms. These include the Th2/Th17 immune responses and epidermal growth factor receptor (EGFR) signaling (20–22). Furthermore, it has been proposed that *STAT3* is a potential target for asthma and SRA therapeutics (23). However, there are also several studies that discount a role for *STAT3* in asthma (24, 25).

*STAT5* is an important regulator of mast cell activity and mediates their proliferation, survival, and homeostasis. Mast cells have been shown to have a key role in the development of asthma, and are implicated in SRA. This suggests that *STAT5* may be involved in asthma through mast cell pathogenesis (8, 26) and there have been several studies supporting such a link (27–31). The association between asthma and the *STAT5b* isoform has been the most well studied relationship to date but a potential role for the *STAT5a* isoform is unclear (14). A previous study by Tsitsiou et al. that evaluated gene expression in patients with severe asthma reported that *STAT3* and *STAT5b* expression levels were 1.59 and 1.62 times higher, respectively in these patients (3). Our study, therefore, aimed to investigate *STAT3* and *STAT5* gene expression in asthma and SRA.

## MATERIALS AND METHODS

Our study was a joint investigation by the National Research Institute of Tuberculosis and Lung Diseases (NRITLD) and the Tarbiat Modares University (TMU) of Tehran-Iran. The cross-sectional study was conducted from 2012–2014 using 13 patients with SRA, 14 non-severe asthma cases, and 30 healthy volunteers. The 2011 international consensus for the definition of severe asthma (2) was used for diagnosis and inclusion of SRA patients. These cases were selected sequentially from the NRITLD asthma clinic. Patients with non-severe asthma were enrolled from the same clinic using criteria outlined by the Global Initiative for Asthma (GINA) (32). Patient involvement was approved by certified pulmonologists. Healthy participants had no history of any compounding disorders at the time of the study. The enrollment of participants was voluntary and signed informed consent forms were collected for each participant. Patient data were kept confidentially and no intervention was applied throughout their clinical management. All stages of the study were approved by the ethical committee of the NRITLD under the code sbmu1.REC.1391.1, dated May 14, 2012.

Following demographic and clinical data gathering, 5 mL samples of peripheral venous blood were obtained from each patient and immediately stored at 4°C. Peripheral blood mononuclear lymphocytes (PBMLs) were separated using a Lympholyte-H (Cedarlane Co., Ontario, Canada) solution during 2 hours. Following the manufacturer’s protocols, RNA was isolated using RNX^Plus^ solution (CinnaGen Co., Tehran, Iran) and the quality and quantity verified using agarose gel electrophoresis and spectrophotometry, respectively.

For each sample, 3 μg of isolated RNA was used to synthezise cDNA using Oligo-dT, random hexamers, and reverse transcriptase enzyme (Fermentas, Thermo Fisher Scientific Co., Waltham, Massachusetts, USA). This was validated using PCR specific to the glyceraldehyde 3-phosphate dehydrogenase (*GAPDH*) gene. qRT-PCR was performed using an Applied Biosystems 7500 sequence detection system (Applied Biosystems, Foster City, CA, USA) and Power SYBR Green I PCR Master Mix (Takara, Japan), according to the manufacturer’s protocol. Primers used for *STAT3* (including all splice variants) and *STAT5A* are indicated in Table 1. The conditions for each qRT-PCR were a preliminary denaturing stage at 95°C for 15 minutes, followed by 40 cycles of denaturation at 95°C for 15 seconds, annealing at 60°C for 20 seconds, and extension at 72°C for 20 seconds. The housekeeping gene of *GAPDH* was used as an endogenous control to normalize *STAT3* and *STAT5A* expression (Table 1).

**Table 1.** The used primers in the study

**Gene**	**Dir**	**Primer Sequence**	**Length (bp)**	**Product length (bp)**	**Efficiency (%)**
GAPDH	F	5′-CCATGAGAAGTATGACAAC-3′	19	115	98.3
	R	5′-GAGTCCTTCCACGATACC-3′	18		
*STAT3* (Var. 1,2,3)	F	5′-AGCAGGAGGGCAGTTTGAGTC-3′	21	241	99.1
	R	5′-TTTAAAAGTGCCCAGATTGCTC-3′	22		
*STAT5A*	F	5′- ACATGTACCCACAGAACCCTGACC-3′	24	239	98.2
	R	5′- CACAACACGACCGCTTCACATTGC-3′	24		

A comparative analysis of the transcript expressioin of *STAT3*, *STAT5A,* and *GAPDH* was performed, using the mean Ct of at least two replicates for each sample. Delta Ct (ΔCt), defined as the difference between the mean Cts of each gene (*STAT3* or *STAT5A*) and the endogenous control (*GAPDH*), was used for further analysis. Statistical differences between the mean ΔCts of the mild asthma, SRA, and control groups were assessed by independent Student’s t-tests and one way ANOVA with a Tamhane’s post hoc test. The primers used for amplification were found to have high efficacy (99.1% for *STAT3* and 98.2% for *STAT5A*), allowing the fold change in transcript levels to be calculated using 2^−ΔΔCt^ methodology (33). Any putative correlation between transcript levels were evaluated using Pearson’s tests. Statistical analyses were performed using the Statistical Package for Social Sciences (SPSS) Version 21.0 (Microsoft, Chicago, IL, USA), with a threshold of significance set at a P-value of 0.05.

## RESULTS

### Participant Characteristics

Table 2 summarizes the demographics of the participants. The mean age was significantly different between healthy individuals and both asthma groups, mainly due to the voluntary nature of the study. Additionally, 90% of the controls were male, while 61% were male for the mild asthma group and 64% for the SRA group. Patient body mass indices (BMIs) were used as a general gauge of the nutritional condition and physical health of participants. This was found to be approximately 26.40 across the three studied groups (P-value = 0.99). Finally, the ethnicities of participants were found to be similar in each of the three groups (60% Fars, 8% Turks, 12% Lores, and 20% Kurds).

**Table 2.** Demographic findings for the participants

	**Severe asthma**	**Asthma**	**Healthy**	**Total**	**P-value**
**Gender**					
Male	8	9	27	44	
Female	5	5	3	13	
Total	13	14	30	57	
**Age**					
Mean (SE)	50.23 (3.80)	54.71 (4.31)	39.17 (1.54)	45.51 (1.80)	<0.001
95% CI	41.95–58.51	45.39–64.4	36.01–42.33	41.89–49.13	
Min.–Max.	21–76	24–80	23–60	21–80	
**BMI**					
Mean (SE)	26.39 (1.25)	26.41 (0.81)	26.49 (0.70)	26.45 (0.50)	0.99
95% CI	23.65–29.14	24.65–28.16	25.04–27.93	25.44–27.45	
Min.–Max.	19.53–35.49	20.05–30.12	18.65–34.09	18.65–35.49	

### Comparison of Transcript Abundance

Student’s t-tests comparing the qRT-PCR data revealed that the transcript expressions of *STAT3* and *STAT5A* were not significantly different between genders (Tables 3 and 4). *STAT3* and *STAT5a* transcript expressions also did not correlate with age of all participants (r=0.42, P-value=0.35 for *STAT3* and r=0.31, P-value=0.67 for *STAT5a*). Furthermore the gene expression (mean ΔCts) of *STAT3* and *STAT5A* were compared by ANOVA statistical tests between cases and control groups.

**Table 3.** The expression difference (ΔCts) of STAT3 in genders and disease groups

	**Mean ± SE**	**95% CI**	**Min.–Max.**	**P-value**
**Gender**				
Male	0.303 ± 0.16	0.02 – 0.66	−2.03 – 2.89	0.47
Female	0.011 ± 0.50	−1.09 – 1.11	−3.00 – 2.55	
**Case/control**				
Severe asthma	0.254 ± 0.47	−0.78 – 1.29	−3.00 – 2.89	0.95
Asthma	0.141 ± 0.40	−0.73 – 1.01	−1.86 – 2.55	
Healthy	0.274 ± 0.16	−0.06 – 0.61	−2.37 – 1.65	
**Total**	0.237 ± 0.16	−0.96 – 0.57	−3.00 – 2.89	

**Table 4.** The expression difference (ΔCts) of STAT5A in genders and disease groups

	**Mean ± SE**	**95% CI**	**Min.–Max.**	**P-value**
**Gender**				
Male	2.88 ± 0.24	2.39 – 3.37	−0.43 – 7.15	0.72
Female	2.72 ± 0.57	1.46 – 3.98	−1.44 – 5.34	
**Case/control**				
Severe asthma	3.64 ± 0.64	2.24 – 5.05	−1.44 – 7.15	0.03
Asthma	3.29 ± 0.35	2.52 – 4.06	1.85 – 5.52	
Healthy	2.33 ± 0.23	1.85 – 2.81	−0.43 – 4.03	
**Total**	2.86 ± 0.22	2.42 – 3.31	−1.44 – 7.15	

**Figure 1.**
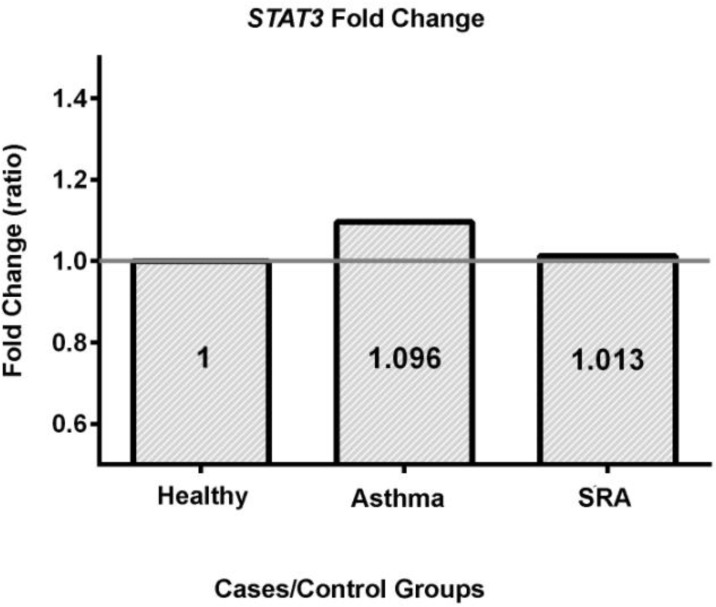
The fold change of gene expression for *STAT3*

Table 3 indicates that there was no significant different between the disease groups in terms of *STAT3* gene expression. Meanwhile, the fold change analysis (2^−ΔΔCt^) showed the expression ratios of asthma and SRA groups against the control group are 1.096 and 1.013 respectively, when the control adjusted to 1 (Figure 1). These findings show the level of *STAT3* gene expression in asthma groupis followed by SRA and control groups.

**Figure 2.**
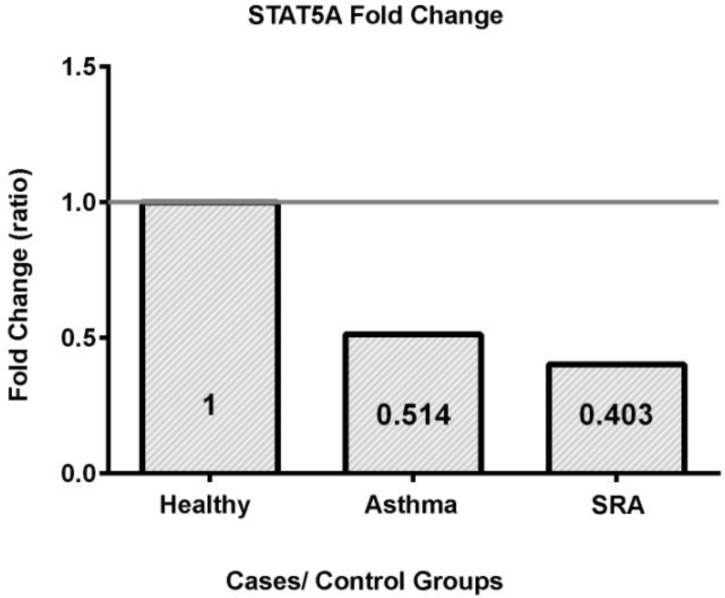
The fold change of gene expression for *STAT5A*

When examining *STAT5a* transcript levels, we found a significant difference between the disease groups (P-value=0.03) (Table 4). A Tukey’s post hoc test suggested that the only significant difference was between healthy and SRA groups (P-value=0.04). The P-value for a putative difference between the mild asthma versus SRA groups, and the asthma versus healthy groups, were 0.83 and 0.15, respectively. Fold change analysis (2^−ΔΔCt^) revealed the highest STAT5a transcript levels were in healthy controls, followed by the mild asthma and then SRA groups (the control group was set to 1.0) (Figure 2).

## DISCUSSION

Considering all participants, the *STAT3* and *STAT5a* gene expressions were not associated with age and significantly different in both sexes. Thus, significant differences of age and gender between the study groups are not confounding factors. Also, similarity in BMI and ethnicity of the groups shows that participants’ physical conditions and genetic backgrounds do not affect the results.

As the steroid regimens and pulmonary function tests of all participants were variable, there was possibility that these factors interfere with our results. To reduce these effects, we applied certain standards restrictedly to prevent bias. For example, the cases were selected if they had diagnostic criteria of GINA (for asthma) and IMI (for SRA) for more than 2 years and had no experience of exacerbation in the 6 months prior to sampling. Furthermore, they were controlled by inhaled corticosteroids which have least systemic effects (34).

### STAT3

As previously mentioned, the role of *STAT3* in the pathogenesis of asthma and SRA is somewhat controversial. Our study revealed that *STAT3* gene expression was not significantly different between the three groups of healthy controls, asthma and severe asthma. This finding is compatible with some reports. For example, Chiba et al. found that *STAT3* had no notable role in the pathogenesis of bronchial allergic asthma (24, 25). Furthermore, the polymorphic relation of asthma and *STAT3* has been denied previously (35) and intracellular flow cytometry of CD4(+)CD161(+) T cells found no differences in phosphorylated *STAT3* levels between patients with asthma and controls (36). On the other hand, many studies signify the *STAT3* role in pathogenesis of asthma and SRA (18–23). It is also believed that airway remodeling may be influenced by *STAT3* (37–40).

Based on our results and previous studies, we hypothesize that, *STAT3* may have some role in the metabolic pathways of asthma, however, it does not seem to be directly involved in the pathogenesis of asthma and SRA. Meanwhile, the controversies in studies may be due to different methods used in each study, the individual role of the three *STAT3* isoforms, an inadequate sample size, or even unknown confounding factors.

### STAT5a

In contrast to *STAT3*, we found a significant difference in the transcript levels of *STAT5a* between the study groups. The *STAT5a* expression in healthy controls was nearly twice of patients with asthma. The groups of patients with SRA had even less transcript, approximately 20% lower than patients with mild asthma. This demonstrates that there is *STAT5a* down regulation in asthma and SRA cases, suggesting that this isoform has a role in the pathogenesis of asthma, particularly its severe form.

The genome wide association studies (GWAS) have found that severe asthma is associated with the 17q21 chromosomal region; the region where codes some proteins like *STAT5a* (6). Furthermore, many evidences signified the role of *STAT5a* in asthma (18, 27), such as that by Stefanowicz et al, who found that *STAT5a* gene expression is decreased in the epithelial cells of airways (41).

Although the exact mechanisms of asthma pathogenesis remain unclear, some studies have suggested possible mechanisms for how *STAT5a* may be involved. These include roles for *STAT5a* in controlling IL-9 expression, the differentiation of Th2, Th9, and Th17 cells (19), the activity of the CD69 receptor and its regulatory role in Th17 cells (42), several mast cell pathways (8, 26), the activity of glucocorticoid receptors (11), lymphocyte proliferation (28), nitric oxide-mediated *STAT5* dephosphorylation (29, 43), and induction of IL-4 producing eosinophils by IL-5 (30). In addition to their individual roles, pathogenesis may be due to a complicated combination of any or all of these factors, or even factors not yet identified. More directly, Burnham et al. found evidence for down regulation of *STAT5* in eosinophil cells during allergic asthma (44), although at least one study found the opposite result. Gernez et al. also showed that there was no difference in intracellular phosphorylated STAT5 levels in CD4(+)CD161(+) T cells between asthmatics and healthy controls (36). As an assumption, the non-different expression of *STAT5* may be due to summation of *STAT5b* up regulation (1.62 times; as Tsitsiou et. al. showed) (3) and *STAT5a* down regulation (>2 times; as we found).

The evidences that *STAT5*, and particularly *STAT5a*, is involved in the pathogenesis of asthma and SRA may present us a new diagnostic or therapeutic horizon. We believe that further study is required to evaluate the importance of *STAT5a* expression as a potential diagnostic tool for SRA. Kabata et al. showed that *STAT5* inhibitors (e.g., Pimozide) can be used to overcome resistance to corticosteroid therapy in patients with asthma (45). The *STAT5* metabolic pathway is therefore a potentially a new target for SRA treatment.

## CONCLUSION

In conclusion, we found no evidence to support the suggestion that *STAT3* is involved in asthma and SRA. Further investigation may provide more information to elucidate its role in respiratory inflammation disorders. Meanwhile, down regulation of *STAT5a* in asthma, and especially SRA, is a notable finding which worth to be considered more in future studies. Using transcriptomic and proteomic methods with higher sample size, the expression study of *STAT5a*, *STAT5b* and total *STAT5* may provide considerable results on their roles in asthma pathogenesis.
